# Freedom and mental health: an overview of the impact of fundamental rights on psychological wellbeing

**DOI:** 10.3389/fpsyg.2025.1470425

**Published:** 2025-07-22

**Authors:** Changiz Mohiyeddini

**Affiliations:** Department of Foundational Medical Studies, Oakland University William Beaumont School of Medicine, Rochester, MI, United States

**Keywords:** autonomy, discrimination, freedom, freedom of speech, human rights, mental health, psychological wellness, social support

## Abstract

Freedom, in its many forms, is a cornerstone of human rights. These include, among others, freedom of speech, freedom of thought, freedom of assembly, and freedom from discrimination. However, these freedoms are not only legal or political constructs but are also deeply embedded in the socio-cultural, religious, political, historical, and hence psychological composition of human beings. Rather than examining all of the many manifestations of freedom at once, this paper aims to explore the impact of a few specific freedoms—including freedom of speech, thought, assembly, movement, and protection from arbitrary arrest—on mental health and wellbeing. The objective of this commentary is to summarize existing evidence on how these freedoms support mental health and to highlight areas where the restriction of freedoms is linked to psychological distress, including anxiety, depression, and post-traumatic stress disorder. Furthermore, it also discusses the debilitating effects of self-censorship, discrimination, lack of access to education, and arbitrary detention on mental health. Despite substantial evidence underscoring the importance of freedoms for mental wellbeing, there remains a pressing need for comprehensive research to understand the complex associations between different aspects of freedom and their individual and collective impact on mental health. The discussion herein aims to provide a foundation for future studies and to inform policy interventions that protect both freedom and psychological wellbeing.

## Introduction

Freedom is a fundamental human right, enshrined in the Universal Declaration of Human Rights (UDHR) (United Nations, 1948). The United Nations General Assembly in 1948 adopted UDHR which outlines many manifestations of freedoms that are essential to preserve human dignity and autonomy. Accordingly, all individuals are, among other essential liberties, entitled to freedom of thought, expression, assembly, and religion. These facets of freedom, to different degrees, are defining characteristics of democratic societies, vital for their civic functioning and democratic as well as peaceful governance and, consequently, contribute to human development (Diamond, 2007; Seen, 1999).

Beyond their importance for democratic societies, these freedoms are pivotal in the mental health and wellbeing of individuals (Dahl, 1998; Diamond, 2007; Diener and Seligman, 2002; Ryan and Deci, 2000). They provide individuals with the autonomy that they need to determine their choices, define, and pursue goals, express their unique identities, and engage with their environment, all of which are crucial for mental health and wellbeing (Hunt et al., 2015; Ng et al., 2012; World Health Organization, 2019).

Although a detailed discussion of each facet of freedom on mental health is beyond the remit of the present paper, it aims to provide a brief overview of studies that have explored the dynamic and complex relationship between freedom and mental health and highlight the enduring need and necessity for further comprehensive research.

## Freedom of speech and mental health

Undoubtedly, freedom of speech is the most widely discussed and studied freedom in the context of defining a society as democratic (Singh et al., 2020). It refers to the right to express one’s opinions and ideas without fear of retaliation or censorship. Empirical studies have consistently demonstrated that this freedom is essential for personal autonomy and self-expression, which are key components of psychological wellbeing (Borba, 2022; González-Luis et al., 2022; Habermas, 1989; Malhotra, 2016; Pinheiro et al., 2015; Rutledge, 2021). Accordingly, individuals who feel they can express themselves freely tend to have higher levels of self-esteem and self-efficacy, and lower levels of anxiety and depression (Krause and Van Tran, 1989; The Lancet, 2016).

Conversely, the fear of expressing one’s opinions due to potential repercussions can lead to significant psychological distress (Førde and Aasland, 2013). This phenomenon, known as self-censorship, can create a sense of isolation and helplessness, which are detrimental to mental health (Hayes et al., 2005; Younis and Jadhav, 2019). The impact of self-censorship on mental health is particularly pronounced in environments where political or social pressures inhibit free expression (Agnew, 1998; Appel, 2022). Studies have demonstrated that individuals in such contexts often experience heightened anxiety, stress, and feelings of powerlessness (Martin et al., 2017).

## Freedom of thought, conscience, and religion

Freedom of thought, conscience, and religion is another central dimension of human rights that substantially impacts mental health and wellbeing (Pargament, 2001). This freedom allows individuals to choose and practice their beliefs without interference or coercion. It is closely linked to personal identity and moral integrity, which are essential for individual growth and personality development (Saroglou, 2011).

Self-determined religious believes, and spiritual practices can provide individuals with a sense of purpose and belonging to a community fostering resiliency and coping mechanisms in times of stress, all of which contribute positively to protect mental health (Koenig et al., 2012). However, restrictions on religious freedom or discrimination based on religious beliefs can lead to marginalization and psychological distress (Jordanova et al., 2015; Pirutinsky et al., 2011). A great deal of research indicates that individuals who face religious discrimination or persecution are at a higher risk of developing mental health issues, including depression, anxiety, and post-traumatic stress disorder (PTSD) (Pargament, 2001; Sharif et al., 2021; Wu and Schimmele, 2021).

## Freedom of assembly and association

The freedom to assemble and associate with others is fundamental to social support, cohesion and community engagement (Fonseca et al., 2018). It allows individuals to come together for common purposes, to pursue shared goals, celebrate common values, share ideas, and advocate for their rights and interests. This freedom is crucial for the development of social support networks, which are vital for mental health and wellbeing (Moustakas, 2023; Putnam, 2000).

Accumulating evidence indicates that social support is significantly associated with a better mental health (Uchino, 2006; Wang et al., 2018). It provides individuals with cognitive, emotional, informational, financial, and practical assistance, which can shield individuals against negative impact of a crisis, buffer against stress and enhance resilience (Callaghan and Morrissey, 1993; Cohen and Wills, 1985). Conversely, restrictions on the freedom of assembly and association can lead to social isolation, loneliness, disenfranchisement, and psychological distress (Xia and Li, 2018). Noteworthy, empirical studies have shown consistently that individuals who are unable to engage in social and community activities due to political or social constraints are more likely to experience feelings of loneliness, anxiety, and depression (Kawachi and Berkman, 2001; Leigh-Hunt et al., 2017).

## Freedom from discrimination

It has long been appreciated that freedom from discrimination is a fundamental human right that underpins the principle of equality. Discrimination based on race, gender, ethnicity, religion, sexual orientation, age, or other characteristics can have profound negative effects on mental health and wellbeing (Gulliford, 2019; Williams et al., 2019). It can lead to social exclusion, stigmatization, and marginalization, which are significant risk factors for mental health disorders (Williams and Mohammed, 2009).

Experiences of discrimination are associated with increased levels of stress, anxiety, and depression (Krieger, 2014; Schouler-Ocak and Moran, 2023). They can also contribute to the development of chronic health conditions, such as hypertension and cardiovascular disease, which are linked to prolonged stress and poor mental health (Dolezsar et al., 2014; Li et al., 2023; Pascoe and Richman, 2009). Addressing discrimination and promoting equality are therefore essential for improving mental health outcomes and protecting the wellbeing of all individuals.

## Right to privacy

Privacy is closely linked to mental health, as it enables individuals to establish boundaries, trust each other, maintain a sense of control, and protect their personal space (Aboujaoude, 2019; Dawson, 2015a; Harris and Orth, 2020; Starr, 1999; Westin, 1967). The right to privacy is fundamental to human dignity and autonomy (Aboujaoude, 2019; Mokrosinska, 2018) as it allows individuals to control their personal information and make decisions about their lives without unwarranted interference (Pew Research Center, 2014).

Invasions of privacy can lead to significant psychological distress (Harris and Orth, 2020). For example, surveillance and data breaches can create feelings of vulnerability, anxiety, and mistrust (Solove, 2008, 2006). Studies have shown that individuals who experience invasions of privacy are more likely to report symptoms of stress, anxiety, and depression (Solove, 2008). Protecting privacy is therefore crucial for mental health and wellbeing, specifically in the digital age.

## Right to education

The right to education has been declared in three international human rights treaties, the Convention on the Rights of the Child (CRC, 1989), the International Covenant on Economic, Social and Cultural Rights (ICESCR, 1966), and the Convention on the Rights of Persons with Disabilities (CRPD, 2006) and is fundamental for personal development and growth, and by extension for social progress (Heckman, 2006; United Nations Children's Fund (UNICEF), 2019).

Education provides individuals with the knowledge and skills needed to achieve their potential and participate fully in society including the labor market (Schultz, 1961; World Bank, 2018). It is also closely linked to wellbeing and healthy aging, as it promotes cognitive development, self-efficacy, and social inclusion (de Mendonça Lima et al., 2023).

Access to education can have a positive impact on mental health by providing individuals with opportunities for personal growth and social engagement. Conversely, barriers to education can lead to social exclusion and psychological distress. Research indicates that individuals who are denied educational opportunities are at a higher risk of experiencing mental health issues, such as depression and anxiety (Patel et al., 2018). Providing and protecting access to quality education is therefore essential for promoting mental health and wellbeing.

## Freedom of movement

It has long been appreciated that the freedom of movement, defined as individuals’ ability to travel within their country and across borders, is essential for personal autonomy. It is closely linked to mental health, as it enables individuals to pursue their goals, access resources such as education or healthcare services, and maintain social connections (Bauder, 2011; International Labour Organization (ILO), 2015; Robertson and Hoffman, 2014; Seen, 1999; Global Education Monitoring Report Team, 2017). Consequently, restrictions on movement, such as travel bans and forced displacement, can have severe debilitating psychological consequences (Cuadrado et al., 2023; Hirani and Richter, 2019) such as feelings of confinement, stress, and helplessness (Mengin et al., 2020; Ochoa-Fuentes et al., 2022). Some insights on the role of freedom of movement in mental health can be borrowed from the literature on combating infectious diseases that show that individuals who are restricted in their freedom of movement are more likely to experience psychological distress and suffer from psychopathological issues (Hawryluck et al., 2004; Hugo, 2002; Jeong et al., 2016). Therefore, protecting and Promoting freedom of movement is essential for safeguarding mental health and wellbeing.

## Freedom from arbitrary arrest and detention

Freedom from arbitrary arrest and detention is crucial for safeguarding individual sense of security and protecting people from governmental overreach and state abuse (De Londras, 2011; Feldman, 1993). Indiscriminate and unjustified detention can have severe psychological consequences, including trauma, anxiety, and PTSD (The Lancet Public Health, 2020). The uncertainty and helplessness associated with arbitrary detention can erode an individual’s sense of security and trust in legal and social institutions (Borschmann et al., 2020; Steel et al., 2002). Over recent decades, empirical studies have shown that the fear of arbitrary arrest can create a pervasive sense of vulnerability and stress impeding individual mental health (Wildeman and Andersen, 2020). Individuals living in environments where arbitrary detention is common often report higher levels of psychological distress and mental health issues (Amnesty International, 2024).

## The interplay between freedoms and mental health: the necessity for further research

Freedom is a complex phenomenon that needs to be put into a psychological framework before it impacts on mental health and wellbeing can properly be understood and explored. The studies noted above provide an important first step in furthering our understanding of associations between facets of freedom and mental health. However, despite abundant work, the relationship between freedom and mental health remains a challenging yet important task for psychological research. Different aspects of freedom can interact in various ways to influence mental health outcomes. Given the significant impact of freedom on mental health and wellbeing, the central conclusion is that there is an eminent need for comprehensive studies that explore the interaction between different aspects of freedom and their influence on mental health. Such studies would provide valuable insights into the mechanisms through which freedom affects mental health and identify potential areas to inform interventions and guide policy developments.

## Conclusion

This review highlights the critical relationship between fundamental freedoms and mental health, illustrating how the ability to exercise freedoms such as speech, thought, assembly, movement, and protection from discrimination directly impacts psychological wellbeing. The evidence shows that these freedoms are essential for maintaining autonomy, social integration, and emotional resilience. Conversely, restrictions on these freedoms are consistently linked to adverse mental health outcomes, including heightened levels of anxiety, depression, and post-traumatic stress disorder.

The analysis underscores the need for further research to explore the mechanisms through which different freedoms interact to affect mental health outcomes. Although the existing literature provides valuable insights, it is clear that gaps remain in our understanding of the specific pathways through which the loss of freedom leads to psychological distress. Addressing these gaps is essential for developing targeted interventions and informing policies that prioritize both the protection of human rights and the promotion of mental health.
